# Hypoxia Induces Autophagy in Human Dendritic Cells: Involvement of Class III PI3K/Vps34

**DOI:** 10.3390/cells11101695

**Published:** 2022-05-19

**Authors:** Sara Monaci, Federica Coppola, Daniela Rossi, Gaia Giuntini, Irene Filippi, Giuseppe Marotta, Silvano Sozzani, Fabio Carraro, Antonella Naldini

**Affiliations:** 1 Cellular and Molecular Physiology Unit, Department of Molecular and Developmental Medicine, University of Siena, 53100 Siena, Italy; sara.monaci@student.unisi.it (S.M.); federica.coppola@student.unisi.it (F.C.); daniela.rossi@unisi.it (D.R.); gaia.giuntini@student.unisi.it (G.G.); filippire@gmail.com (I.F.); 2 Cellular Therapy Unit, South-East Tuscany Blood Establishment, University Hospital, 53100 Siena, Italy; g.marotta@ao-siena.toscana.it; 3 Laboratory Affiliated to Istituto Pasteur Italia-Fondazione Cenci Bolognetti, Department of Molecular Medicine, Sapienza University of Rome, 00185 Rome, Italy; silvano.sozzani@uniroma1.it; 4 IRCCS Neuromed, 86077 Pozzilli, Italy; 5 Department of Medical Biotechnologies, University of Siena, 53100 Siena, Italy; fabio.carraro@unisi.it

**Keywords:** hypoxia, dendritic cell, autophagy, PI3K, SAR405

## Abstract

Hypoxia is a component of both physiological and pathological conditions, including inflammation, solid tumors, and lymphoid tissues, where O_2_ demand is not balanced by O_2_ supply. During their lifespan, dendritic cells (DCs) are exposed to different pO_2_ and activate different adaptive responses, including autophagy, to preserve their viability and functions. Autophagy plays multiple roles in DC physiology. Very recently, we demonstrated that hypoxia shapes autophagy in DCs upon their differentiation state. Here, we proposed a role for PI3Ks, and especially class III PI3K/Vps34, that could be relevant in hypoxia-induced autophagy, in either immature or mature DCs. Hypoxia inhibited mTOR phosphorylation and activated a pro-autophagic program. By using different pharmacological inhibitors, we demonstrated that hypoxia-induced autophagy was mediated by PI3Ks, especially by Vps34. Furthermore, Vps34 expression was enhanced by LPS, a TLR4 ligand, along with the promotion of autophagy under hypoxia. The Vps34 inhibitor, SAR405, abolished hypoxia-induced autophagy, inhibited pro-survival signaling and viability, and increased the expression of proinflammatory cytokines. Our results underlined the impact of autophagy in the maintenance of DC homeostasis at both cell survival and inflammatory response levels, therefore, contributing to a better understanding of the significance of autophagy in DC physiology and pathology.

## 1. Introduction

Hypoxia is a component of both physiological and pathological conditions. In physiology, the partial oxygen pressure (pO_2_) in healthy tissues is between 3% and 9% relative to that present in the atmosphere at sea level (≈20% O_2_) [1]. Interestingly, in lymphoid organs, the pO_2_ is even lower, being usually below 2.5% [2]. In pathology, hypoxia is a hallmark of inflammatory processes, solid tumors, and other situations, where the O_2_ demand is not balanced by O_2_ supply [3]. Indeed, the pO_2_ drops to ≈2% and sometimes to 0.1%, representing almost an anoxic condition. We and others have shown that hypoxia regulates the development and the functions of both innate and adaptive immune cells [4,5,6]. Dendritic cells (DCs) are professional antigen presenting cells and during their lifespan are exposed to different pO_2_ as they traffic between different compartments, characterized by different pO_2_ gradients [7,8]. They represent a bridge between innate and acquired immunity and their dysregulation may lead to an amplification of inflammation, loss of tolerance or establishment of immune escape mechanisms [9]. 

We have previously shown that hypoxia can profoundly affect DC behavior, by activating pro-apoptotic or pro-autophagic responses, depending upon their maturation state [6,10]. Of interest, autophagy is not the only adaptive cellular mechanism induced by hypoxia, but is also fundamental for proper DC differentiation and function [11]. mTOR is an autophagy-associated protein and it is inhibited by many signals, such as nutrient starvation and hypoxia [12]. mTOR can regulate autophagy through direct phosphorylation of ULK1 [13,14]. The binding of the ULK complex to Beclin1–Vps34–PI3K originates a pre-autophagosomal structure. Then, the phagophore elongation occurs depending on two ubiquitin-like conjugation cascades, including the Atg5-Atg12 and the microtubule-associated light chain 3 (MAP-LC3/Atg8/LC3) conjugation systems. The lipidated form of LC3B, LC3B-II, cooperates with SQSTM1/p62, an adaptor molecule with multiple functions, which stimulates the turnover of poly-ubiquitinated protein aggregates. This is followed by the fusion with endosomes and lysosomes to develop autolysosomes where lysosomal degradation can occur [15].

Several signaling pathways are involved in the control of autophagy. For example, the PI3K/AKT/mTOR-mediated signaling pathway can inhibit autophagy [16] and is implicated in the metabolic reprogramming of immune cells, including DCs [17]. In contrast, the Ras/Raf/ERK signaling pathway plays a crucial role in promoting autophagy [18]. Autophagy is also stimulated by the dissociation of Bcl-2 from Beclin-1 upon post-translational modification of Bcl-2 and constitutive Bcl-2 phosphorylation [19].

The phosphoinositide-3kinase (PI3K) family comprises the following three different classes: class I, II, and III. Among them, two isoforms of class I, namely p110γ and PI3Kδ, and class III PI3K (Vps34) are the most expressed and well characterized in immune cells [20]. We and others have previously reported the role of class I PI3Ks in DC cell migration and survival [6,21,22]. Recently, a highly selective and potent Vps34 inhibitor, SAR405, was reported to affect autophagy [23]. More importantly, Vps34 was shown to control selective DC subsets under homeostatic conditions [24]. This observation suggests that PI3Ks, including Vps34, may be crucial for the induction of autophagy in DCs exposed to hypoxia.

Here, we report the role of hypoxia in the inhibition of mTOR phosphorylation and activation of a pro-autophagic program involving PI3Ks, mostly Vps34. These results may contribute to a better understanding of the significance of autophagy in DC biology as a modulator of cell survival and inflammation in hypoxic microenvironments.

## 2. Materials and Methods

### 2.1. Reagents

RPMI 1640, fetal bovine serum (FBS), penicillin/streptomycin and L-glutamine were purchased from Euroclone, Devon, UK. Fycoll was obtained from Cederlane Labs and Percoll from Amersham Bioscience, Pittsburgh, PA, USA. Recombinant human granulocyte macrophage colony stimulating factor (GM-CSF) and interleukin-13 (IL-13) were purchased from ProSpec TechnoGene, East Brunswick, NJ, USA. All the reagents contained <0.125 endotoxin units/mL, as checked by the Limulus amebocyte lysate assay (Cambrex, East Rutherford, NJ, USA). LPS from Escherichia coli strain 026:B6 was obtained from Sigma-Aldrich, Milano, Italy. Wortmannin, LY 294002, U0126 and BAY 11-7082 were purchased from Tocris Biosciences, Bristol, UK. SAR405 was purchased from CliniSciences, Nanterre, France. Baf A1 was obtained Enzo Life Sciences, Plymouth Meeting, PA, USA.

### 2.2. Human Monocyte-Derived DC Preparation and Culture Conditions

The study was reviewed and approved by the Ethical Committee of Azienda Ospedaliera Universitaria Senese and University of Siena (CAVSE 17022020). The participants provided their written informed consent. Human monocyte-derived DCs were generated from anonymous buffy coats (South-East Tuscany Blood Establishment, AOUS, Siena) as previously described [21]. DCs were exposed either to normoxia or hypoxia (2% O_2_, corresponding to a pO_2_ ~ 14 mmHg) in the workstation InVIVO O_2_ 400 (Ruskinn, Pencoed, UK). Where indicated, DC terminal maturation was induced by adding LPS (100 ng/mL). In some experiments, the cells were treated with Wortmannin (5 µM), LY294002 (50 µM), SAR405 (10 µM), BAY 11-7082 (10 µM), U0126 (5 µM) Baf A1 (100 nM) 6 h before the end of the experiment/LPS treatment. At the indicated times, cells were harvested for further analysis, as described below.

### 2.3. Western Blot

The DCs were lysed in a RIPA buffer (Cell Signaling Technologies, Danvers, MA, USA) in the presence of a cocktail of protease inhibitors (Sigma-Aldrich, St. Louis, MO, USA). Equal amounts of total proteins were loaded onto SDS-PAGE gel and blotted onto a nitrocellulose membrane (BIO-RAD, Hercules, CA, USA). The membranes were incubated overnight, at 4 °C, with the following primary antibodies: HIF-1α (BD Biosciences, San Jose, CA, USA); phmTOR, mTOR, phULK1 ser 757, SQSTM1/p62, phAKT and AKT, Mcl-1, Vps34, Beclin-1, Atg5, phERK, α-Tubulin (Cell Signaling Technologies, Danvers, MA, USA); ULK1 and LAMP1 (Santa Crutz Biotechnology, Dallas, TX, USA) and β-actin (Sigma-Aldrich). Anti-mouse IgG HRP and anti-rabbit IgG-HRP (Cell Signaling Technologies, Danvers, MA, USA) were used as secondary antibodies. Images were acquired by ChemiDoc™ MP System and blot quantification was performed by using Image Lab software (BIO-RAD, Hercules, CA, USA).

### 2.4. RNA Isolation, Extraction and RT-qPCR

EuroGOLD^TM^Trifast reagent (Euroclone, Devon, UK) was used to extract the total RNA from the harvested cells. Then, cDNA was synthesized using the iScript^TM^cDNA Synthesis Kit (BIO-RAD, Hercules, CA, USA). RT-qPCR was performed using iTaq^TM^SYBR Green Supermix (BIO-RAD, Hercules, CA, USA). mRNA expression levels of BNIP3, IL-6, IL-12 and TNFα were determined by the MiniOPTICON^TM^ System and the analysis was performed with an iQ5^TM^ Optical System Software (BIO-RAD, Hercules, CA, USA). Relative quantification was carried out using the 2^−ΔΔCT^ method [25] and β-actin as the housekeeping gene. 

### 2.5. Immunofluorescence, Lysotracker and Confocal Microscope Analysis 

Immunofluorescence and Lysotracker staining and confocal microscope analysis were performed as previously described [10]. Briefly, for immunofluorescence staining, the DCs were plated on sterile chamber slides (Nunc Lab-Tek) stimulated or not with LPS and incubated for 24 h, under either normoxic or hypoxic conditions. When indicated, the DCs were treated with SAR405 in the last 6 h of incubation. After fixation (in cold methanol) and permeabilization (with HEPES/Triton), the cells were blocked (with 10% goat serum). Then, the cells were incubated overnight in a humidified chamber, at 4 °C, with the following primary antibodies: LAMP1 (Santa Crutz Biotechnology, Dallas, TX, USA); LC3B and β-actin (Cell Signaling Technologies, Danvers, MA, USA). The following day, nuclear staining was performed using DAPI (Calbiochem, San Diego, CA, USA) and cells were incubated with Cy2 (green) or Cy3 (red) (Jekson laboratories) conjugated secondary antibodies. For Lysotracker staining, the DCs were seeded on an 8-well coverglass slide (Sarstedt, Germany), stimulated or not with LPS and exposed for 24 h to either normoxic or hypoxic conditions. When indicated, the DCs were treated with Wortmannin or SAR405 in the last 6 h of incubation. After incubation, the cells were labeled by the Lyso-ID Green Detection Kit (Enzo Life Sciences, Plymouth Meeting, PA, USA) and DAPI was used for nuclear staining. The assembled slides were imaged with an LSM-510 META confocal microscope (Carl Zeiss, Oberkochen, Germany) and the fluorescence intensity was measured by ImageJ software.

### 2.6. Cell Viability Analysis 

Cells were plated in 96-well plates, stimulated with LPS, treated or untreated with SAR405 and incubated under hypoxia for 48 h. Fluorescein diacetate was diluted in acetone (1 mg/mL, 1:200) and the working solution was added to each well (100 μL/well). Cells were incubated for 30 min in the dark at 37 °C. Then, fluorescence was measured (λex: 494 nm and λem: 518 nm) using a microplate reader (FLUOstar Optima, BMG Labtech, Durham, NC, USA). The viability index was calculated as the percentage of the ratio between the treated group and control.

### 2.7. Statistical Analysis 

The data are shown as the mean ± SEM of at least 3 independent experiments. Statistical analyses were made with Graph-Pad Prism (San Diego, CA, USA). An analysis of variance (ordinary one-way ANOVA) and an unpaired two-tailed Student’s t-test were used and a difference of *p* ≤ 0.05 was considered to be statistically significant (* *p* ≤ 0.05)

## 3. Results

### 3.1. Hypoxia Inhibits mTOR Phosphorylation and Induces Autophagy in DCs

**Figure 1:**
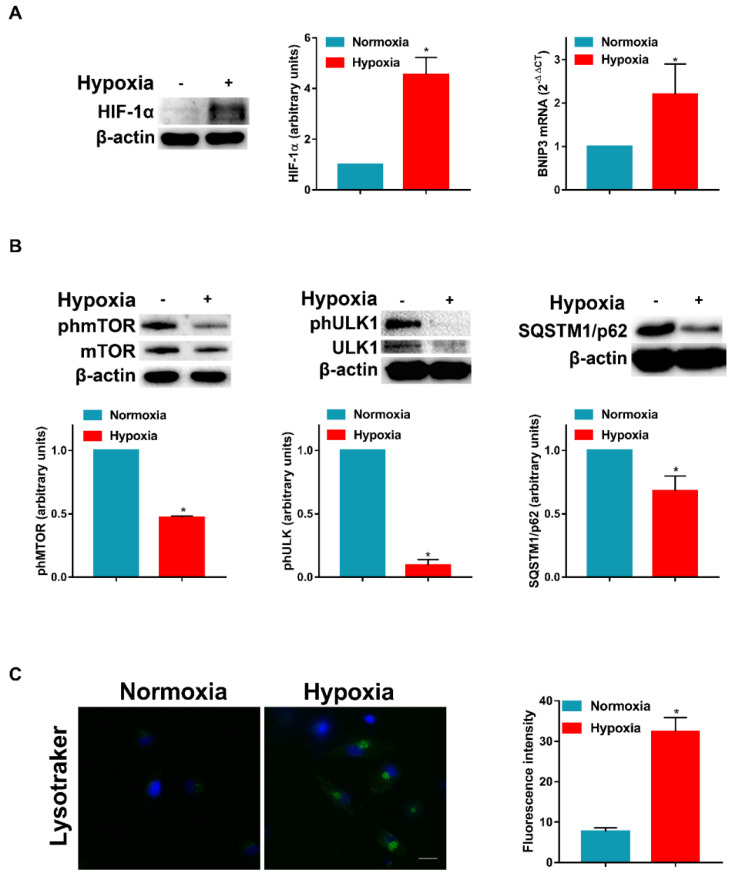
**Hypoxia inhibits mTOR phosphorylation and induces autophagy in DCs.** (**A**) HIF-1α protein levels by Western blotting and BNIP3 mRNA by RT-qPCR analysis, after 24 h exposure to normoxia and hypoxia. (**B**) phmTOR, mTOR, phULK1, ULK1 and SQSTM1/p62 protein levels by Western blotting. (**C**) Detection of acidic/lysosomal compartments by Lysotracker and confocal analysis (Scale bar: 15 µm). All blots shown are representative of at least three independent experiments and β-actin was used as loading control and housekeeping gene for RT-qPCR analysis. Asterisk indicates statistically significant differences (*p* ≤ 0.05; *n* = 3).

We have previously stated that hypoxia modulates the signaling pathways associated with DC survival and apoptosis [6]. More recently, we have demonstrated that hypoxia shapes autophagy in LPS-activated DCs [10]. Thus, we decided to investigate the mechanism by which hypoxia may affect the autophagic flux in DCs. First, we exposed DCs to a pO_2_ of 140 mmHg (normoxia) or 14 mmHg (hypoxia) for 24 h and determined the HIF-1α protein level and the expression of BNIP3 mRNA, which is strictly controlled at the transcriptional level by HIF-1α. As expected, at a pO_2_ of 14 mmHg, DCs expressed a significant increase in HIF-1α protein level, which was paralleled by a significant enhancement of BNIP3 mRNA expression (Figure 1A). Of note, BNIP3 is one of the main regulators of hypoxia-induced autophagy in several cell types [26] and it mediates the hypoxia-induced inhibition of mTOR [27]. Figure 1B clearly shows that the phosphorylation of mTOR was significantly reduced in hypoxic immature DCs. This was paralleled by the decreased phosphorylation of ULK1 at Ser757, which resulted in the initiation of the autophagic process [28]. The promotion of the autophagic flux by hypoxia was unequivocally confirmed by the reduced expression of SQSTM1/p62 protein levels. In addition, in Figure 1C, confocal microscopy showed a significant rise of Lysotracker staining, which supported the hypothesis that hypoxia promoted autophagy in DCs.

### 3.2. Hypoxia-Induced Autophagy Is Mediated by PI3Ks

**Figure 2:**
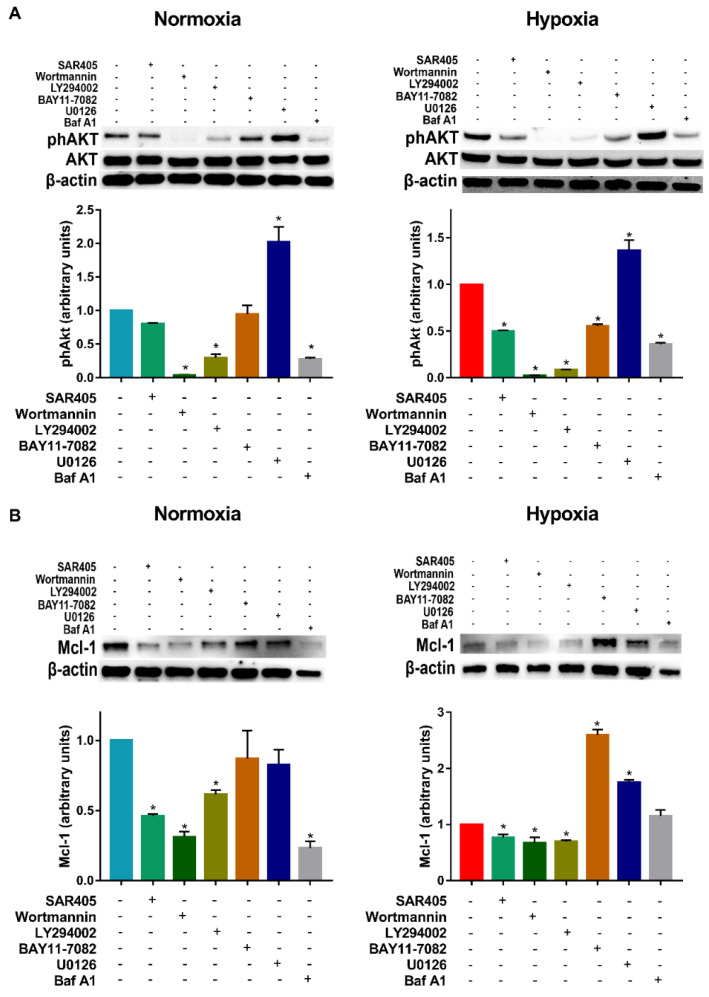
**Effects of pharmacological inhibitors on phAKT and Mcl-1 protein expression.** (**A**) phAKT protein levels in DCs exposed to normoxia and hypoxia for 24 h and treated or untreated in the last 6 h with SAR405, Wortmannin, LY294002, BAY11-7082, U0126 and Baf A1, as determined by Western blotting. (**B**) Mcl-1 protein levels in DCs exposed to normoxia and hypoxia for 24 h and treated or untreated in the last 6 h with SAR405, Wortmannin, LY294002, BAY11-7082, U0126 and Baf A1, as determined by Western blotting. All blots shown are representative of at least three independent experiments and β-actin was used as loading control. * indicates statistically significant differences (*p* ≤ 0.05; *n* = 3).

Since hypoxia promoted autophagy in DC, we decided to investigate the mechanisms potentially involved. To this end, DCs were exposed to hypoxia in the presence of several pharmacological inhibitors of pathways, which are classically or potentially associated with DC survival and autophagy. These include the Vps34 inhibitor SAR405, the irreversible and reversible pan PI3K inhibitors Wortmannin and LY294002, the NFkB/IKK inhibitor BAY11-7082, the MAPK inhibitor U0126 and, finally, the widely accepted autophagic inhibitor Baf A1. Since in previous reports we clearly depicted the role of PI3K/AKT in DC survival under hypoxia [6], we initially focused the experiments on the phosphorylation of AKT. As expected, under normoxia, while SAR405 was unable to reduce AKT phosphorylation, Wortmannin and LY294002 were significantly effective (Figure 2A). BAY11-7082 was also unable to reduce AKT phosphorylation. Furthermore, U0126 increased AKT phosphorylation. Of note, treatment with the autophagic inhibitor Baf A1 resulted in the inhibition of AKT phosphorylation. A similar pattern was observed when the DCs were exposed to hypoxia. To test whether these inhibitors could affect the expression of proteins that are associated with DC survival, and, eventually, with autophagy, we next analyzed the protein levels of Mcl-1. The latter is an anti-apoptotic Bcl-2 family member that has been proposed to promote the survival and differentiation of DCs [29]. As shown in Figure 2B, PI3K inhibitors, including SAR405, significantly reduced Mcl-1 expression under either normoxic or hypoxic conditions in DCs. 

**Figure 3:**
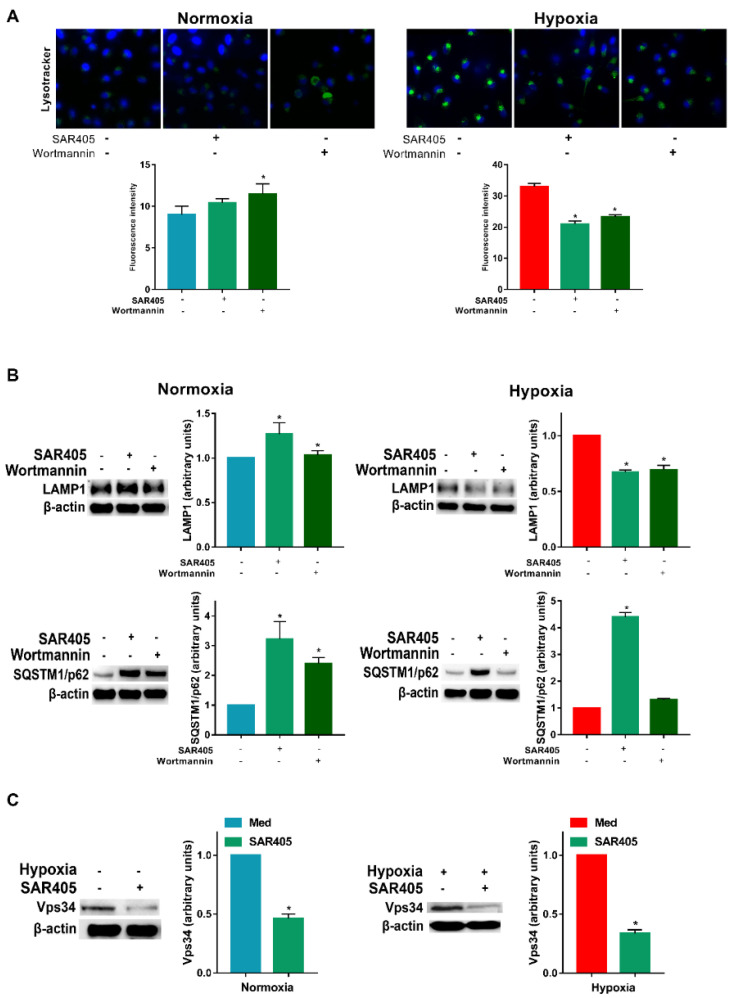
**Hypoxia-induced autophagy is mediated by PI3Ks.** (**A**) Detection of acidic/lysosomal compartments by Lysotracker and confocal analysis (scale bar: 15 µm) and (**B**) LAMP1 and SQSTM1/p62 protein levels in DCs exposed to hypoxia for 24 h and 48 h, respectively, and treated in the last 6 h with SAR405 and Wortmannin. (**C**) Vps34 protein levels in DCs exposed to hypoxia for 24 h and treated in the last 6 h with SAR405. All blots shown are representative of at least three independent experiments. β-actin as loading control. * indicates statistically significant differences (*p* ≤ 0.05; *n* = 3).

Since PI3Ks appear to be involved in the modulation of autophagy, we next determined their role in the autophagic flux using inhibitors. As shown in Figure 3A, under normoxia, we did not observe a significant reduction in the Lysotracker staining assay. However, in hypoxic DCs, the evident staining was significantly reduced by either SAR405 or Wortmannin. A similar result was obtained for LAMP1 expression, whose protein levels were significantly reduced only under hypoxia (Figure 3B). Regarding SQSTM1/p62, its expression was significantly increased by both SAR405 and Wortmannin under normoxia. More interestingly, under hypoxia, only SAR405 was capable to induce a strong enhancement of SQSTM1/p62. 

SAR405 is recognized as the elective inhibitor of class III PI3K, which is considered one of the key molecules in autophagy [23]. Accordingly, Vps34 expression was inhibited upon SAR405 treatment under both normoxia and hypoxia (Figure 3C). 

### 3.3. LPS Enhances Vps34 Expression and Promotes Autophagy in Hypoxic DCs

**Figure 4:**
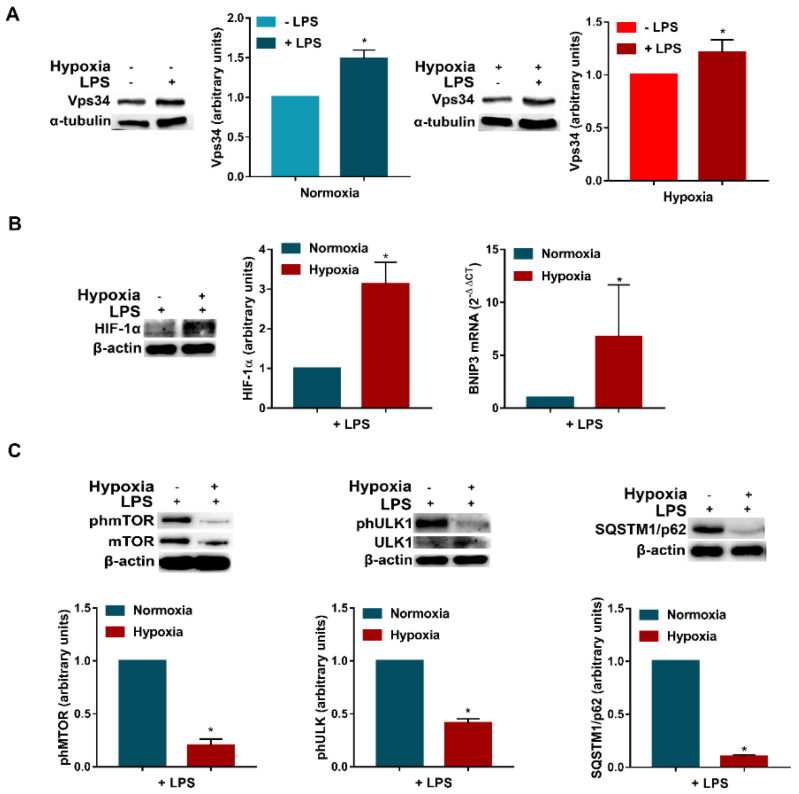
**LPS enhances Vps34 expression and promotes autophagy in hypoxic DCs.** (**A**) Vps34 protein levels in DCs exposed to normoxia and hypoxia, in the presence or not of LPS, for 24 h. (**B**) HIF-1α protein levels, as determined by Western blotting and BNIP3 mRNAs as determined by RT-qPCR analysis, respectively, in DCs stimulated with LPS and exposed to normoxia and hypoxia for 24 h. (**C**) phmTOR, mTOR, phULK1, ULK1 and SQSTM1/p62 protein levels by Western blotting. All blots are representative of at least three independent experiments. β-actin was used as loading control and as housekeeping gene for RT-qPCR analysis. Asterisk indicates statistically significant differences (*p* ≤ 0.05; *n* = 3).

**Figure 5:**
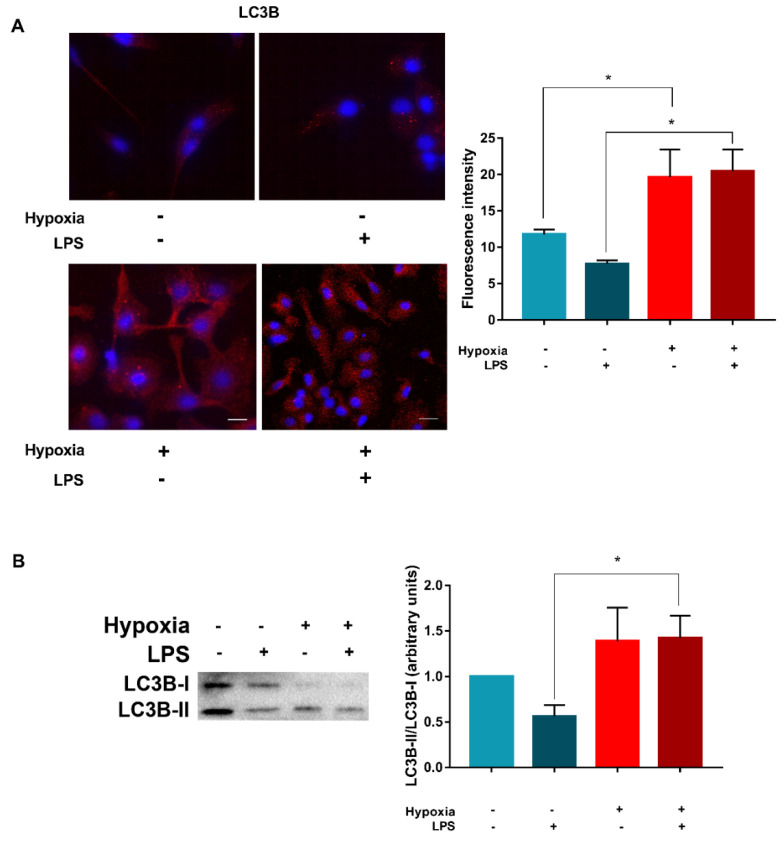
**Hypoxia and LPS affects LC3B**. (**A**) LC3B levels after 24 h stimulation without or with LPS and exposure to normoxia and hypoxia, as determined by confocal microscopy analysis (scale bar: 15 µm). (**B**) LC3I/LC3II conversion by Western blotting. All blots shown are representative of at least three independent experiments and β-actin was used as loading control. Asterisk indicates statistically significant differences (*p* ≤ 0.05; *n* = 3).

The above results indicate that PIK3s, especially Vps34, are involved in hypoxia-induced autophagy in DCs. Thus, further experiments were conducted to evaluate whether the promotion of autophagy in LPS-activated DCs could be associated with Vps34 expression. As shown in Figure 4A, when the DCs were treated with LPS under either normoxia or hypoxia, we observed a significantly higher expression of Vps34, suggesting a potential involvement of class III PI3K. Even in this case, hypoxic treatment increased the accumulation of HIF-1α (Figure 4B). This effect was associated by the upregulation of BNIP3 mRNA expression, indicating a more pronounced hypoxic signature in the presence of LPS. Of note, similarly to immature DCs, hypoxia significantly reduced mTOR phosphorylation (Figure 4C), and thus the promotion of autophagy. This effect was associated with the downregulation of ULK1 phosphorylation at Ser 757 and the reduction in SQSTM1/p62 expression. The induction of autophagy in LPS-treated DCs by hypoxia was further confirmed by confocal analysis. As shown in Figure 5A, confocal analysis revealed that hypoxic LPS-treated DCs present a significantly higher expression of LC3B. This was confirmed by Western blot analysis, showing a significant increase in LC3B-I to LC3B-II conversion (Figure 5B).

### 3.4. Inhibition of Class III PI3K/Vps34 Abolishes Autophagy in Hypoxic LPS-Treated DCs

**Figure 6:**
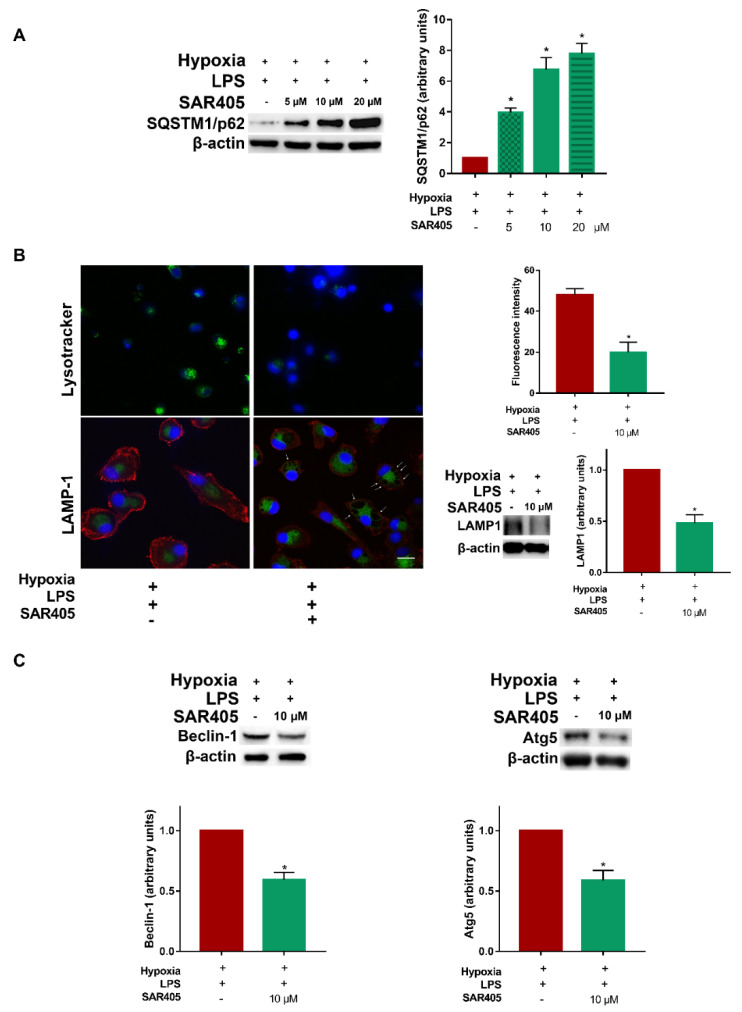
**Inhibition of class III PI3K/Vps34 abolishes autophagy in hypoxic LPS-treated DCs.** (**A**) SQSTM1/p62 protein levels as determined by Western blotting in DCs stimulated with LPS, exposed to hypoxia for 24 h and treated or untreated in the last 6 h with SAR405 (5 µM, 10 µM, 20 µM). (**B**) Detection of acidic/lysosomal compartments by Lysotracker and LAMP1 confocal analysis (scale bar: 15 µm) in LPS stimulated DCs, exposed to hypoxia for 24 h and treated or untreated in the last 6 h with SAR405; LAMP1 protein levels as determined by Western blotting in LPS stimulated DCs, exposed to hypoxia for 48 h and treated or untreated with SAR405. (**C**) Beclin-1 and Atg5 protein levels as determined by Western blotting in LPS stimulated DCs, exposed to hypoxia for 24 h and 48 h, respectively and treated or untreated in the last 6 h with SAR405. All blots shown are representative of at least three independent experiments and β-actin was used as loading control. * indicates statistically significant differences (*p* ≤ 0.05; *n* = 3).

As class III PI3K/Vps34 appeared to be clearly involved in autophagy in DCs, we performed a series of experiments aimed at further defining the mechanism by which the inhibition of Vps34 may affect the autophagic flux in LPS-treated DCs under hypoxia. To this end, we first conducted dose-response experiments to evaluate the effects of SAR405 on the expression of SQSTM1/p62. As shown in Figure 6A, SAR405 induced a significant upregulation of SQSTM1/p62 protein levels, indicating the inhibition of autophagy. This effect followed a dose-response pattern, which was as follows: 5 µM concentration was already capable of affecting SQSTM1/p62 expression, which was more evident at 10 and 20 µM concentrations. To further determine the inhibitory effects of SAR405 on autophagy, we next selected a variety of autophagy indicators, which are related to different steps of the autophagic flux. At first, we evaluated the impact of SAR405 on lysosome acidification by confocal microscopy. As shown in Figure 6B, 10 µM SAR405 induced late endosome-lysosome swelling, as determined by using Lysotracker and anti-LAMP1 antibodies. The inhibitory effect of SAR405 on LAMP1 was further confirmed by Western blot analysis, where we detected a significant downregulation of LAMP1 protein levels. The inhibitory effects of SAR405 were also significant for Beclin-1 and Atg5 (Figure 6C), thus, indicating that class III PI3K plays a crucial role in the activation of autophagy in hypoxic LPS-treated DCs. 

### 3.5. SAR405 Affects Survival and Inflammatory Cytokine Expression in Hypoxic LPS-Treated DCs

**Figure 7:**
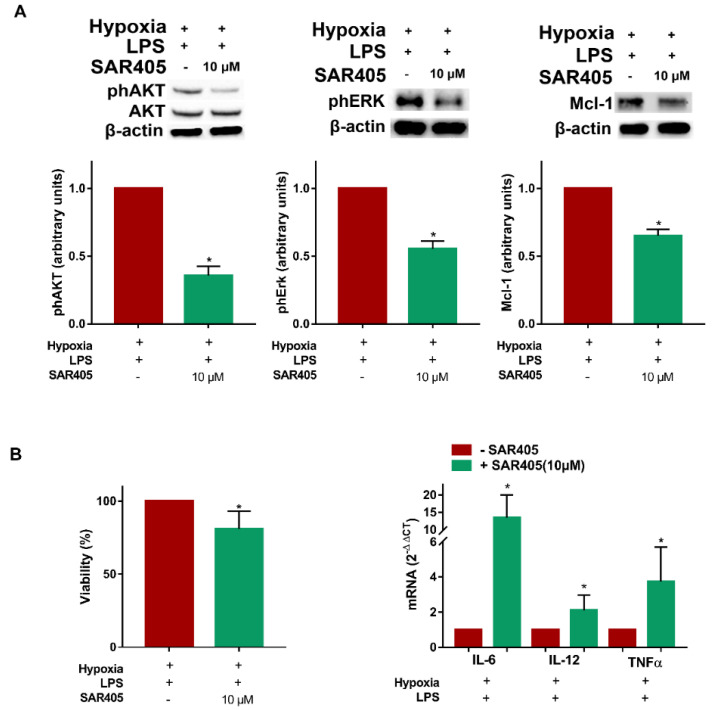
**SAR405 affects survival and inflammatory cytokine expression in hypoxic LPS-treated DCs.** (**A**) phAKT, phERK and Mcl-1 protein levels as determined by Western blotting in LPS stimulated DCs, exposed to hypoxia 24 h and treated or untreated in the last 6 h with SAR405. (**B**) Cell viability (48 h), and IL-6, IL-12 and TNFα mRNAs (24 h), as determined by fluorescein diacetate assay and RT-qPCR analysis, respectively, in LPS stimulated DCs, exposed to hypoxia and treated or untreated in the last 6 h with SAR405. All blots shown are representative of at least three independent experiments and β-actin was used as loading control. β-actin was used as a housekeeping gene for RT-qPCR analysis. * indicates statistically significant differences (*p* ≤ 0.05; *n* = 3).

The above results indicate a tight correlation between autophagy and hypoxic DC maturation with the involvement of Vps34. Thus, we next determined whether SAR405 could affect the signaling pathways involved in DC survival and/or autophagy. Figure 7A clearly shows that, in hypoxic LPS-treated DCs, AKT phosphorylation was significantly reduced by SAR405 treatment. In addition, we observed a reduction in ERK phosphorylation and Mcl-1 expression, which are associated with the modulation of DC autophagy and survival [29]. The role of class III PI3K inhibition on hypoxic LPS-treated DCs was finally confirmed by the results regarding DC viability. As shown in Figure 7B, the treatment of DCs with SAR405 resulted in a significant reduction in the percentage of viable DCs, as determined by the fluorimetric analysis. Surprisingly, but in accordance with previous reports, SAR405 treatment resulted in a higher expression of the pro-inflammatory cytokines IL-6, IL-12 and TNF-α [30]. The overall results indicate that the inhibition of autophagy by SAR405 resulted not only in reduced DC survival, but also in the enhanced expression of proinflammatory cytokines, thus, underlining the impact of autophagy in the modulation of inflammatory responses.

## 4. Discussion

The impact of hypoxia on innate immune cell physiology has been extensively reported [4]. In the present manuscript, we showed, for the first time, that pharmacological inhibition of PI3Ks, and in particular of Vps34, affected the autophagic flux induced by hypoxia in DCs. Indeed, treatment with SAR405, a highly specific Vps34 inhibitor, resulted in reduced DC survival with regard to their final maturation induced by the MD-2/TLR4 ligand LPS. In addition, we observed that SAR405 under hypoxia treatment increased the expression of the pro-inflammatory cytokines IL-6, IL-12 and TNF-α in LPS-activated DCs.

We and others have previously demonstrated that hypoxia significantly affects DC functions, with important physiological and pathological implications in the immune response [5,21,31,32]. In DCs, autophagy is involved in their maturation steps, including antigen presentation and cytokine production [11]. Recently, we reported that hypoxia induces autophagy in DCs, with particular regard to LPS-activated DCs, indicating a protective effect by TLR-4 activation against hypoxic insult [10]. The purpose of this manuscript was to investigate the mechanisms that could be potentially involved in the induction of autophagy by hypoxia in either immature or activated DCs. In agreement with previous reports, hypoxia induces significant upregulation of HIF-1α and BNIP3 mRNA expression [27]. Thus, our hypoxic condition (2% O_2_), which represents the average pO_2_ present in the lymphoid organs, in inflammation and in neoplastic tissues was capable of activating hypoxic downstream signaling [33,34,35]. 

The inhibitory effect of hypoxia on the upstream regulator of autophagy mTOR has been widely reported in several cell types [12,28]. mTOR can regulate autophagy through direct phosphorylation of ULK1, varying on the phosphorylation sites [13]. Indeed, ULK1 phosphorylation at serine 757/758 results in the inhibition of the autophagic program [14]. Accordingly, the levels of phosphorylated mTOR and phosphorylated ULK1(Ser 757) were downregulated in both immature and fully maturated hypoxic DCs. This was associated with decreased SQSTM/p62 and increased LC3B protein levels and LC3B-II/LC3B-I ratio, as well as with a higher number of acidic/functional lysosomes, thus, documenting the activation of the autophagic flux under hypoxia [36,37,38]. It is widely accepted that most cells keep low basal autophagy to survive under normal circumstances and that autophagy may be further implemented by different stimuli, including hypoxia [39,40]. In addition, previous reports indicate that hypoxia promotes autophagy, thus, resulting in prolonged cell survival [41,42]. The anti-apoptotic Bcl-2 family member, Mcl-1, has been proposed to promote the survival and differentiation of DCs [43]. In addition, previous studies report PI3K-dependent upregulation of Mcl-1 in innate immune cells [44]. Accordingly, in the present manuscript, the expression of Mcl-1 was reduced by the pharmacological inhibitor of PI3K/AKT (Wortmannin), as well as by the Vps34 inhibitor (SAR405). 

Previous reports indicate that both class I and class III PI3Ks are involved in the promotion of autophagy in several cell types, including DCs [20,45]. In agreement with this hypothesis, in our study, both Wortmannin and SAR405 were able to inhibit hypoxia-induced autophagy in DCs. This was documented by a lower number of acidic/functional lysosomes and by an inhibition of LAMP1. Of note, previous reports described that LAMP1 is expressed constitutively at high levels by all cells, including DCs [46]. LAMP1 is crucial in the maintenance of the integrity of the lysosomal membrane in various cells [47] and LAMP1 knockdown is associated with decreased lysosome integrity, increased ROS production, and decreased cell viability [48]. More interestingly, only SAR405, but not Wortmannin, was able to increase SQSTM/p62 under hypoxia, confirming the specific role played by class III PI3Ks in DC autophagy [24]. Earlier studies reported that SQSTM/p62 plays an essential role in the formation and the autophagic degradation of aggregosome-like induced structures, which might be critical for regulating host defense [49]. In addition, TLR4-mediated autophagy in macrophages is SQSTM/p62-dependent [50]. Indeed, TLR stimulation modulated the autophagy flux, in particular upon the stimulation of primary DCs by LPS [51]. More recently, we showed that hypoxia shapes autophagy in LPS-treated DCs [10], confirming the cytoprotective effects caused by autophagy in DC survival under hypoxic conditions. In the present study, we identified one of the potential mechanisms involved in hypoxia-induced autophagy in LPS-treated DCs. Indeed, we demonstrated that SAR405 treatment resulted in an inhibition of the autophagic flux and, more interestingly, in decreased DC viability. This was associated with the diminished activation of pathways or molecules that correlated to DC survival [52]. Indeed, SAR405 inhibited the phosphorylation of AKT and ERK in hypoxic LPS-treated DCs. Previous reports demonstrated that hypoxia induces the activation of AKT, thus, resulting in an adaptive response and protective role against cell death/apoptosis [53]. In addition, AKT has been proposed as a critical regulator of DC lifespan, with important implications in DC-based tumor vaccines [54]. Earlier publications reported that ERK is also activated upon hypoxia [55]. More recently, we showed that hypoxia enhances ERK phosphorylation in DCs, either in the presence or in the absence of LPS [10]. Of note, it has been recently described that ERK signaling stimulates autophagy to protect the renal cells from hypoglycemia-induced cell death [56]. 

It has been previously reported that, in innate immune cells, the upregulation of the anti-apoptotic Bcl-2 family member, Mcl-1, is dependent upon PI3K [44]. Accordingly, in the present manuscript, we observed that SAR405 inhibited Mcl-1, confirming the impact of autophagy, and specifically of Vps34, in DC cell survival. In addition to its effects on cell survival/death, autophagy plays another important role in balancing inflammation in innate immunity [57]. Indeed, previous reports has shown that the inhibition of autophagy resulted in a disruption of inflammatory regulation [58]. In addition, earlier observations described that Sar405 treatment resulted in an increased expression of both pro- and anti-inflammatory cytokine in bone marrow-derived DCs [30]. In agreement with these previous articles, we show here that Sar405 treatment resulted in increased upregulation of IL-6, IL-12 and TNF-α. This could also be particularly relevant in cancer treatment, as it has been recently reported that inhibition of Vps34 reprograms cold into hot inflamed tumors, thus, improving anti-PD-1/PD-L1 immunotherapy in melanoma and colorectal cancer mouse models [59]. In this regard, future studies, including the targeting of Vps34 by RNA interference, are certainly needed to further understand the possible involvement of class III PI3K/Vps34 in regulating DC inflammatory responses in a hypoxic context, similar to that observed in the tumor microenvironment. Preliminary results in our laboratory, obtained by flow-cytometry analysis, indicate that Vps34 inhibition by SAR405 increases the percentage of CCR7-positive DCs by three times, when treated with LPS under hypoxia (data not shown). This suggests, once again, an important implication of autophagy for DC migration and, in particular, for the homing of mature DCs to lymph nodes [11]. In conclusions, our data indicate that PI3Ks, and in particular Vps34, may play an important role in the autophagic and pro-survival responses of DCs exposed to hypoxia, especially upon LPS activation. Since autophagy is crucial for DC orchestration of the immune response, our study may provide the base for new future therapeutic applications, including immunotherapy, where DC functions need to be not only preserved but also improved. 

## Data Availability

The raw data supporting the conclusions of this article will be made available by the authors, without undue reservation.
